# Galvanic Action within the Mouth. Its Relation to Dental Surgery

**Published:** 1878-01

**Authors:** Geo. Watt


### GALVANIC ACTION WITHIN THE MOUTH. ITS RELATION TO DENTAL SURGERY.

BY GEO. WATT.

*Written for the Ohio State Dental Society.*

Coming from the medical into the dental profession, I fancied that dentists had paid less attention to chemistry than to other sciences collateral with Dental Surgery. This fancy had much to do in guiding my own thoughts and efforts in the endeavor to contribute my share toward the advancement of dental science. And whether or not this fancy were founded on fact, I certainly noticed less distinctness of thought, in reference to the chemistry of the mouth, than I found on other subjects. And this was especially true, in regard to the chemical results of galvanic action in the mouth. Hoping mainly to aid my immediate classmates in mastering this subject, I made it the theme of an inaugural thesis, when a student in the Ohio College of Dental Surgery. (See "Intrastomatic Galvanism," in Watt's Chemical Essays.)

Within the last few years, much thought has been bestowed on this subject, especially by two or three members of the profession. The industry manifested by the brethren, referred to, is highly commendable, and is, I hope, duly appreciated by us all. With reluctance, let me say, that a closer attention to elementary principles would have saved much labor, and would have guided to more correct conclusions. Finding still a great lack of clear thinking on galvanic action within the mouth, without attempting to criticise any one, I will offer a few spontaneous thoughts, without reference to authorities and text books, and with but little effort toward systematic instruction, aiming to write just as I would talk to my most familiar personal friends; for what else am I now doing? And if the first person singular number should appear too prominent, I hope you will regard egotism as not more assuming than we-gotism, and that you will remember, without frequent mention of the fact, that every statement herein, is simply "according to my humble opinion."

Then, for galvanic action within the mouth, and its relation to Dental surgery.

Galvanism is modified electricity. An apparatus for setting in motion, or developing this condition of the electric fluid, is called a galvanic battery. Batteries are of two kinds, essentially, and each kind is subject to various modifications. One is composed of two perfect conductors of galvanic electricity, and an imperfect one. This is the more common variety. The elements of the other kind are one perfect, and two imperfect conductors. This is less common, and as it is, neither by intention nor accident placed within the mouth, it may be dismissed from present consideration. Only the first variety is of practical moment to the dental surgeon, and bearing in mind its constituent elements, remember also that unless the imperfect conductor acts chemically on one of the perfect ones, galvanism is not manifested. If it acts on both, it must act unequally, or no current is established. For illustration of these principles, think of the most common batteries. Zinc and copper are called perfect conductors ; while the acidulated water, or saline solution, is an imperfect one. Though the metals differ in conducting power, they are all called perfect conductors, as they relate to galvanism. In the zinc and copper battery, just referred to, the liquid acts on both metals; but, as it acts with more energy on the zinc than on the copper, galvanic action is manifested and the greater the difference in the action on the two plates, other things being equal, the greater is the galvanic manifestation. Hence, if the copper is replaced by a metal which totally resists the action of the fluid, there is a gain in the power of the battery. In Groves', and some other batteries, platinum is used instead of copper.

If the two plates of a battery, say zinc and copper, are placed in the exciting fluid, they assume opposite electric states, when not in contact with each other, or when not united with a metallic conductor; but there is only static electricity. But when brought in contact, either directly or by an intervening metallic conductor, the electricity is set in motion, and what is known as a galvanic circle is established. Such an electric current is a potent agent in chemical decomposition, but it has its limits, to be hereafter noticed.

Decomposition, by a galvanic current, is called electrolysis. A substance which can be so decomposed, is called an electrolyte. A conductor conveying a galvanic current into an electrolyte, is called an electrode. In practical chemistry, the electrodes, usually wires, convey the galvanic current into the electrolyte, generally contained in a vessel or reservoir at a greater or less distance from the battery. The preparation of A. J. Watt's crystal gold, is a beautiful illustration of this. But nothing analogous to such electrolysis ever occurs within the mouth, and if some of my brethren had duly appreciated this, they would have been saved much labor, with less confusion in their teachings. While decomposition is taking place in the receptacle where the electrodes terminate, to exactly the same degree is the liquid in the battery decomposed, provided that the battery is perfect, for all parts of a galvanic circle equal each other in quantity and power. This electrolysis, or decomposition, and this only, is analogous to that which may and does occur in the mouth. Had this been always borne in mind, the time and trouble of many experiments, which throw no light on this subject, might have been avoided.

It has been intimated that electrolysis has its limits, only liquids can be electrolyzed; but how often do we hear of tooth substance or dentos, whatever that may be—being decomposed by electrical currents! Water is readily electrolyzed, but ice, never. And not all liquids are electrolytes. Some, as alcohol, will not conduct galvanic currents at all, and, of course cannot be decomposed by such currents. And, further, only binary compounds, that is, such compounds as contain but two elements are decomposed by galvanic currents. Let not this be misunderstood. A liquid may be able to conduct galvanic currents, while it holds in solution a number of binary compounds. The current will decompose there in the ratio of their chemical equivalents. For example, in the mouth is found water, chloride of sodium, chloride of potassium, and sometimes ammonia, sulphuretted hydrogen, etc., each containing but two elements, and each, therefore, subject to electrolytic decomposition.

Thus far reference is had to true electrolysis—true galvanic decomposition, but the chemical action thus liberated must not be overlooked. And these secondary actions must not be confounded with electrolysis, as has been far too common, I fear, by very many of my professional brethren. Very much of the chemical results of galvanic action is due to the affinities of the elements thus set free, which being presented in their nascent state, have the increased energy incident to that condition. If a tooth is in any degree disintegrated or corroded, as a result of galvanic action, the phenomenon is due to this secondary action, and never directly to electrolysis.

I regard the subject as greatly simplified, when this is clearly understood. Confusion of thought has resulted from an unprofitable discussion of the direction of the galvanic current in the battery, and along the electrodes. By confining our thoughts to the battery alone, we gain clearness of view. All galvanic action within the mouth is analogous to what occurs here. To aid the memory, let us examine the action within the ordinary battery. If a strip of pure zinc and another of copper are placed in a liquid composed of sulphuric acid and water, and these strips are allowed to touch each other, or are united by an intervening metallic conductor, something takes place. The oxygen of the water unites with and corrodes the zinc; but when water yields oxygen, a corresponding equivalent of hydrogen is liberated. This gas being but slightly, if at all, soluble in water and unable to unite with either of the metals, escapes in bubbles, but only at the surface of the copper, if the battery is perfect. In the entire list of elements, oxygen and hydrogen are at opposite extremes of the electric scale. Oxygen is always more highly electronegative than any other element, while hydrogen is more electropositive than any other. But, as we have seen, oxygen goes to the zinc in the battery, and this because the zinc is positive, or in an electric state opposite to that of the oxygen. And the hydrogen goes to the copper, because it is negative, a state opposite to that of the hydrogen. Hydrogen is probably a metal. Now remember that all the metals take the same direction, when separated from the corrosive elements by electrolysis; and the corrosives, such as chlorine, sulphur, fluorine, etc., follow the example of oxygen. This brief statement, retained in the memory, affords a key to almost any supposable case of electrolytic decomposition.

To remember the direction of the positive current in the galvanic circle, we need only recall the fact that the zinc or corroded metal is positive. The current passes from this through the liquid to the copper, or uncorroded metal, along this to the electrode or connecting metal, and back to the zinc. The negative current, equal in power and velocity, traverses, simultaneously, the opposite direction; and this is the reason that telegrams can be sent in opposite directions, at the same time on a single wire.

A singular attempt has been made to apply a knowledge of the direction of the positive current to practical use in filling teeth. I allude to the suggestion that different metals be so arranged, in the same cavity, that a positive electric current will pass from the tooth substance to the filling, as if it were the current striking the tooth that destroys it. An old hunter used to show me how smoothly polished he kept the front sight of his rifle by looking along it. The two propositions are equally sound. But after all, guiding the positive current from the tooth to the filling, is only guiding the equally powerful negative current from the filling to the tooth.

Circumstances being equal, the quantity of electricity set in motion by a galvanic battery is directly in proportion to the surface excited. A pure zinc plate four inches square will give off sixteen times the quantity afforded by a similar plate an inch square.

Another elementary fact, often ignored, is, that the decomposing powers of batteries, other things being equal, vary inversely as the square root of the distances between the plates. When the plates are one inch apart the decomposing power is twice as great as when they are four inches, and four times as great as when they are sixteen. And in like manner, when they are a fourth of an inch apart, the power is double that of one inch, and is increased four-fold when they are but a sixteenth of an inch. An imperceptible distance (apparent contact) is many times less than a sixteenth of an inch, yet the increase of power is governed by the same law. The practical electrician sees daily illustrations of this, on account of the impurities of the zinc of commerce. Small particles of iron are often diffused through the zinc, which, with the latter metal, form an infinitesimal series of minute batteries of wonderful decomposing power, as is manifested by the great abundance of bubbles caused by the escaping hydrogen. This is an illustration of true electrolysis, as good as any known to electro-chemical science. The ultimate elements are separated in accordance with their relative electric properties, each going to the metal whose electric state is the opposite of its own.

Now suppose we place a plate of impure, commercial zinc, in a liquid containing not only water, but chloride of sodium, sulphuretted hydrogen, and ammonia. Here are four binary compounds, liquid capable of conducting galvanic currents, and acting on the particles of both the zinc and iron, but with the greater energy on the zinc. The water will be electrolyzed, and it is easy to remember that its oxygen goes to and corrodes the zinc, while its hydrogen goes to the iron, and escapes in gas bubbles, unless it there finds something with which to combine. But the salt is also electrolyzed, its chlorine being a corrodent, follows the oxygen, while the sodium goes to the iron. Sulphur goes with the oxygen,--its hydrogen of course to the iron.

When we reflect that these corrosive elements are brought to the zinc, in obedience to electric attraction rather than to chemical affinity, we can readily see that their corrosive action need not be, and is not all spent in corroding the zinc. When liberated they are free to obey the laws of affinity. But it is well known that nascent chlorine takes hydrogen from almost any compound containing it. The disinfecting and bleaching properties of chlorine are mainly due to this fact. It will, therefore, take hydrogen from water, forming hydro-chloric acid, and this at the zinc. Ammonia composed of hydrogen and nitrogen, if present, is also electrolyzed; and hydrogen being always electropositive, of course the nitrogen has to go to the zinc, and as it there, in its nascent state, finds oxygen also nascent, combination takes place, nitric oxyd being first formed, which in the presence of air and water, is rapidly changed to nitric acid. It is on this principle only that the white variety of dental caries is ever found as a result of galvanic action within the mouth.

It might prove unprofitable to notice all the modes of producing galvanic action within the mouth. If I can say enough to awaken an interest in the subject, and to develop clearness of thought in reference to it, among my fellow members, this to me, laborious effort, will reap its full reward.

I fear that a few of my brethren misapprehend sometimes, and suspect the presence of galvanic currents in the mouth when the conditions necessary to their production are not present. For example, some have inferred that gold and platinum, in contact in the mouth, will give such results; but how can this be unless the fluids of the mouth corrode at least one of the metals? which is not likely to take place, unless in highly abnormal conditions. Again, when a cavity is partly filled with tinfoil, and the plug is finished with gold, there is often pain and irritation, on change of temperature within the mouth. This is usually spoken of as a result of galvanic action, which it can hardly be, unless the plug leaks. The pain is ordinarily due to what, in the text books, is called thermo-electricity. Also, when two metals are placed in the mouth, and one is corroded by the buccal fluid, if they are not in contact, and if there is no better conductor connecting them than the fluid itself, the metals assume opposite electric states, but no current is established and decomposition of the fluid does not take place. The condition is that of static electricity. But if the metals touch each other or both touch the mucus membrane of the mouth, a regular circle is developed and decomposition occurs in proportion to the quantity of galvanism thus set in motion. A gold plug and one of any base metal may constitute such battery, and let us suppose these plugs are in adjacent teeth, and in contact with each other at a given point, or both in contact with the gum at the necks of the teeth, the binary compounds in buccal fluid must be decomposed, but how? Water and soluble chlorides are present in all mouths. Then, of water, the oxygen, and of the salts, the chlorine go to the base metal, and the hydrogen of the water, and the metals of the chlorides go to the gold. But we have already seen that nascent chlorine takes hydrogen from compounds containing it, hence, hydrochloric acid must be formed at the base metal. And this is why we so often see the most common variety of dental caries about the margins of the base metal plugs; this decay being always the result of the action of hydrochloric acid.

Rape is a crime unknown in chemistry, and there is no social restriction among the elements demanding that one sex shall do all the courting. Hence, when the hydrogen reaches the gold, it takes chlorine from compounds containing it, as promptly as itself is captured by the chlorine at the other side of the battery. But by this combination we have hydrochloric acid again, and this is why we so often find the same variety of decay around the gold filling. At the other side much of the force of the chlorine, as well as of the acid, is spent in corroding the base metals, while neither the hydrogen nor the acid can attack the gold; but all the force of the acid is spent on the the tooth; and thus the tooth with the gold plug is, in such cases, more corroded than that with the base metal, which leads some to the conclusion that the base metal is the better filling. A similar arrangement, with the same results may be made by a gold plate and a base metal plug, as well as by other means which may be omitted from the present discussion.

When a gold plug leaks, there is apt to be galvanic action. The organic matter of dentine is a conductor, and in this case, represents the corroded or zinc side of the battery. Hydrochloric acid decay occurs in such cases, when the buccal fluids are normal, but when ammonia is present, we may have white decay co-operative with it, owing to the formation of nitric acid by electrolysis of the ammonia.

The "almalgam question" would be entirely omitted here, if faithfulness to science would permit. Its general bearings, of course, lie beyond the range of this paper. Only as amalgams are factors in galvanic action within the mouth, will they be noticed and even in this aspect but briefly.

The word "amalgam," always implies that mercury is one of its constituents. Probably the first one used in filling teeth was composed of silver and mercury. That most extensively used for many years, contained tin, in addition. This may be taken as a type of the race, without stopping to discuss special formulas. Satan is the devil still, even if clothed in the garb of an angel of light; and the ass brayed when he tried to roar, although covered with the skin of a lion.

Very few will claim I think that amalgam plugs are pure alloys, in the same sense that brass is an alloy of zinc and copper. The alloy prepared ready for the mercury is made by commingling or fusing together, metals without any reference to their chemical equivalents, and in some cases by using metals which will not combine to form a true alloy. Then the mercury is added by guess work, and its excess is squeezed out till the operator thinks he has squeezed it sufficiently tight, and guesses about enough free mercury has escaped; and, although in amalgamating there is much more oxidation in some cases than in others, he guesses that will make no difference, even if it should change the proportions of his metals, which he guessed were about right at the start; and he guesses he can fill with amalgam, even if he cannot with gold.

The scientific metallurgist will tell you, that while there is something of the alloy in your amalgam, there is, at the same time, much of the mechanical mixture. At best it is analogous to commercial zinc, as an exciter of galvanic action in a corrosive fluid. Tin has less affinity for oxygen than either silver or mercury. The infinitesmal particles of tin become negative, while the corresponding particles of the other metals are rendered positive. As the distances are imperceptible, the decomposing power is very great in proportion to the surface excited. Thus an amalgam filling has, within itself, all that is necessary to electrolyze the binary compounds of the buccal fluids. It may also act as the positive or corroded side of a battery, when in the proper relation with a gold plug or plate; but its principle galvanic power is as already described.

But this paper, much too long, must be brought to a close. I hope the hints herein set forth will aid my fellow members to clearness of thought in reference to this important subject. Allow me to say that I have not used, in this paper, the latest chemical nomenclature. I am more familiar with the old. Probably a majority of my fellow members are too. It will thus better accord with my previously written essays, should it be published with the proceedings of this meeting. And as technical terms are avoided as far as practicable, I hope my younger brethren, who are more familiar with the new nomenclature, will have but little difficulty in following the thoughts set forth. Allow me also to say that this paper is written for this society at the request of some of its most active and experienced members.

